# Impact Assessment of an Innovative Integrated Care Model for Older Complex Patients with Multimorbidity: The CareWell Project

**DOI:** 10.5334/ijic.4711

**Published:** 2020-05-22

**Authors:** Maider Mateo-Abad, Ane Fullaondo, Marisa Merino, Stefano Gris, Francesco Marchet, Francesca Avolio, Elisabetta Graps, Mario Ravic, Mario Kovac, Vanesa Benkovic, Ranko Stevanovic, Antoni Zwiefka, Daniel Davies, Silvia Mancin, Antonella Forestiero, Panos Stafylas, Mayte Hurtado, Marco d’Angelantonio, Signe Daugbjerg, Claus Duedal Pedersen, Reinhard Hammerschmidt, Veli Stroetmann, Lierni Azkargorta, Anna Giné, Dolores Verdoy, Myriam Soto-Gordoa, Joana Mora, Javier Mar, Itziar Vergara, Esteban de Manuel Keenoy, on behalf of the CareWell project group

**Affiliations:** 1 Kronikgune Institute for Health Services Research, Barakaldo, Basque Country, ES; 2 Health Services Research on Chronic Patients Network (REDISSEC), Barakaldo, Basque Country, ES; 3 Osakidetza Basque Health Sevice, Tolosaldea Integrated Health Care Organization, Tolosa, Basque Country, ES; 4 Azienda ULSS 1 Dolomiti, Local Health Authority, Feltre, Belluno, IT; 5 Agenzia Regionale per la salute e Sociale – Regione Puglia, Bari, Puglia, IT; 6 Health Technology Assessment – UNIT, Regional Strategic Agency for Health and Social Affair (AReSS), Bari, IT; 7 Ericsson Nikola Tesla d.d., Zagreb, HR; 8 Dept. of Control and Computer Engineering, Faculty of Electrical Engineering and Computing, University of Zagreb, Zagreb, HR; 9 Croatian Society for Pharmacoeconomics and Health Economics, Zagreb, HR; 10 Department of Health, LSV Marshal Office, PL; 11 Powys Teaching Health Board, Bronllys Hospital, Bronllys, Powys, UK; 12 Arsenàl.IT, Veneto’s Research Centre for eHealth Innovation, Treviso, IT; 13 HealThink, Thessaloniki, GR; 14 Health Information Management S.A. – HIM S.A., MAST European Economic Interest Group, Brussels, BE; 15 Centre for Innovative Medical Technology, Odense University Hospital, Odense, DK; 16 Empirica Communication and Technology Research, Bonn, DE; 17 Biodonostia Health Research Institute, Donostia, Basque Country, ES; 18 Osakidetza Basque Health Service, Debagoiena Integrated Health Organisation, Mondragon, Basque Country, ES; 19 Biodonostia Health Research Institute, Primary Care Group, Donostia, Basque Country, ES

**Keywords:** integrated care, elderly, multimorbidity, patient empowerment, ICT

## Abstract

**Objectives:**

To evaluate the impact in terms of use of health services, clinical outcomes, functional status, and patient’s satisfaction of an integrated care program, the CareWell program, for complex patients with multimorbidity, supported by information and communication technology platforms in six European regions.

**Data Sources:**

Primary data were used and the follow-up period ranged between 8 and 12 months.

**Study design:**

A quasi-experimental study, targeting chronic patients aged 65 or older, with 2 or more conditions – one of them necessarily being diabetes, congestive heart failure or congestive obstructive pulmonary disease. The intervention group received the integrated care program and the control group received usual care. Generalized mixed regression models were used.

**Data collection:**

Data were obtained from individual interviews and electronic clinical records.

**Principal Findings:**

Overall, 856 patients were recruited (475 intervention and 381 control). In the intervention group, the number of visits to emergency rooms was significantly lower, and the number of visits to the general practitioners and primary care nurses was higher than in the control group.

**Conclusion:**

The CareWell program resulted in improvements in the use of health services, strengthening the role of PC as the cornerstone of care provision for complex patients with multimorbidity.

## Introduction

In aging populations, multimorbidity (two or more chronic diseases in the same person [1,2] is very common [3,4,5]. Patients with multimorbidity have complex health and social needs, are at risk of being admitted to the hospital or residential care home and require a wide range of interventions [6].

To satisfy the needs of these patients and their families, new innovative integrated care models are needed. To be effective, they should have primary care as the cornerstone of care [7]; effective integration between care levels [8,9]; empowered patient’ and carers/families’ [8]; and it should be patient-centered. The use of information and communication technology (ICT) platforms could facilitate and improve communication promoting patient empowerment and home support [9,10]. This innovative interoperability should increase effectiveness, efficiency, and equity [11,12].

The evaluation of such programs is challenging, since multiple dimensions [13] should be taken into account, such as clinical outcomes, use of services and patient’s satisfaction, organizational processes and systems, community wellbeing, or population health. Published results ranged from notable improvements in all measures [14,15] to lack of significant changes in comparison with usual care [16,17]. So properly evaluated interventions based on innovative integrated care programs are necessary for the decision-makers to propose and implement optimal organizational models [18,19].

The aim of the CareWell project is to implement and to assess the effectiveness of an integrated care program based on the coordination between health providers, home-based care, and patient empowerment, supported by ICT-based platforms. A large range of dimensions were assessed through a quantitative and a qualitative approach. In this work, we will focus and report the quantitative evaluation of clinical effectiveness, physical functional status, use of health services, patient’s satisfaction outcomes, although organizational aspects, economic impact and safety has been evaluated also for the overall project.

## Theory and Methods

### Study design

This is a quasi-experimental study based on the CareWell project targeting chronic patients aged 65 or older with multimorbidity (Trial Registration: ClinicalTrials.gov Identifier- NCT03042039). The intervention group received the new integrated care program and the control group received usual care [20].

### Study population

The CareWell program targeted patients with the following inclusion criteria: 1) age 65 or older; 2) a minimum of two chronic diseases, with at least one of them being chronic obstructive pulmonary disease (COPD), diabetes mellitus, or chronic heart failure (CHF); 3) classified as complex by their care system, considering, among others, severity, increased vulnerability, complex health needs, high risk of hospitalization, and/or intensive use of resources; and 4) able to understand the study instructions and requirements, either independently or with the assistance of their caregiver. Exclusion criteria were: 1) patients with an active cancer diagnosis; 2) individuals after an organ transplant; 3) patients receiving dialysis; 4) candidates for palliative care (with life expectancy of less than one year); 5) patients diagnosed with AIDS; or 6) living in private care homes.

The program was piloted in six European regions: Basque Country (Spain), Zagreb (Croatia), Lower Silesia (Poland), Veneto (Italy), Puglia (Italy), and Powys (UK). Potential participants were identified, depending on the pilot site, using a different source: reviewing the electronic healthcare records (EHR), mining hospital or national databases, or at clinical review routine encounters. Based on these target populations a convenience non-probabilistic sampling method was used. Candidates were informed about the nature and the objectives of the study and those willing to participate, signed the informed consent sheets. Intervention and control groups were set up in all Pilot sites, with the exception of Powys that had just the intervention group. The intervention group patients entered into the CareWell integrated care model. Patients in the control group received care as usual.

### Data collection

Sociodemographic and lifestyle information, physical functional status and patient’s satisfaction were collected at baseline (February 2015) and in the follow up period (8-12 months). Clinical measurements were collected in face to face individual encounters conducted by a trained nurse. During the follow-up period use of services and other control variables were collected from different sources, including administrative databases, electronic health records and questionnaires.

### Intervention

The CareWell integrated care model is based on two main elements: 1) care coordination and communication between health providers and 2) patient empowerment and home-based care; all supported by ICT-based platforms. Eight Integrated care related Service Procedure areas were identified: transition support, self management, patient follow up, care manager, multidisciplinary team, stratification, specialist consultant and multidisciplinary case meetings. Twelve relevant ICT tools for Integrated Care support were defined: Electronic prescription, Messaging clinician and Patients, Electronic Health Record, Interconsultation, Call Center, Virtual Conference, Personal Health Folder, Nurse Information System, Educational Platform, Collaborative Platform, Telemonitoring and Multichannel Centre. Pilot sites, based on a self-assessment exercise, identified their improvement areas and decided on the specific Service Procedure and Technological Adaptation to implement, according to their baseline situation, context. The ICT architecture supporting care coordination and patient empowerment was defined and implemented. In summary, the intervention resulted in: a) Care Coordination element: better communication between healthcare professionals (primary and secondary care), better definition of care manager role, improved information sharing between healthcare professionals via central storage of data and definition of shared care plans and smooth transition support by facilitating information sharing after hospital discharge using ICT systems. b) Patient Empowerment and Home Support element: promotion patient and caregiver empowerment through access to health-related educational material, patients can access or enter clinical information and book appointments via distinct ICT tools, messaging between healthcare professionals and patients/caregivers and remote monitoring of patients’ health status. Descriptive material is provided as complementary documents (Table S1) and an in-depth description of the care pathways defined at each site and their deployment strategies can be found in the CareWell Project website [20]. Once implemented, patients’ follow-up period ranged between 8 and 12 months.

### Variables

Primary outcome measures were use of services, clinical control of the examined conditions, physical functional status and patient’s satisfaction. Baseline and confounding factors such as sociodemographic and lifestyle variables were also measured.

Use of health services was assessed recording number of contacts with health care providers (general practitioners (GPs), nurses, specialists, and others), number of contacts with social services, number of contacts with the hospital and duration of hospitalizations, and visits to emergency rooms (ER).

Regarding clinical control, diagnosed chronic conditions included in the Charlson Comorbidity Index (CCI) were documented and other health-related variables were also recorded: Body Mass Index (BMI), blood pressure (mmHg), heart rate (bpm), oxygen saturation (%), blood glucose (mg/dl), HbA1c (%), and creatinine (mg/dl). Presence of depressive symptoms was examined using the short form of Geriatric Depression Scale (GDS) [21]. Physical functional status was assessed using the Barthel Index [22,23]. Sociodemographic and lifestyle characteristics including gender, age, level of education, smoking habits, and use of devices (mobile phones and personal computers (PC)) were also collected. Finally, patient’s satisfaction was assessed using the Policy Innovation Research Unit’s (PIRU) questionnaire on the user experience of integrated care [13,24].

### Statistical analysis

Categorical variables are presented using frequencies and percentages, n (%). Differences between groups were examined using the χ^2^ test. Continuous variables with a normal distribution are presented as means with standard deviation (SD); differences between groups were established using Student’s *t*-test. Continuous variables with non-normal distribution are presented as median and first and third quartiles (Q1, Q3), and differences were examined using nonparametric Wilcoxon rank-sum test. Pre and post differences for categorical variables were calculated employing the McNemar’s test for paired data.

To assess the effect of CareWell intervention in an intention to treat basis, longitudinal generalized mixed models were used, including the pilot sites as random effect. These models were adjusted by the follow-up period, and other confounding factors. Linear multivariate regression was performed for continuous outcomes, and multivariate logistic regression, for discrete outcomes. The effect of the intervention was studied by the care group-by-follow-up period-interaction effect. The results shown indicate the significance of this interaction term by its corresponding p-value, and its coefficient estimation for 12 months follow-up period. All the analyses were performed using the free statistical software R program v. 3.2.1.

### Ethics approval and consent to participate

The project was evaluated and approved by the local ethical committees before its implementation, when appropriate (Basque Country: CEIC Euskadi No. PI2014200; Poland: No. 1/NT/2015; Croatia: 01.3602/2-2014; Puglia: extension of ASL Lecce No. DG1000/2484; Powys and Veneto: did not required ethical approval as the project was classified as service evaluation by their ethics committee). All participants provided their written informed consent.

## Results

### Characteristics of the study population

**Figure 1:**
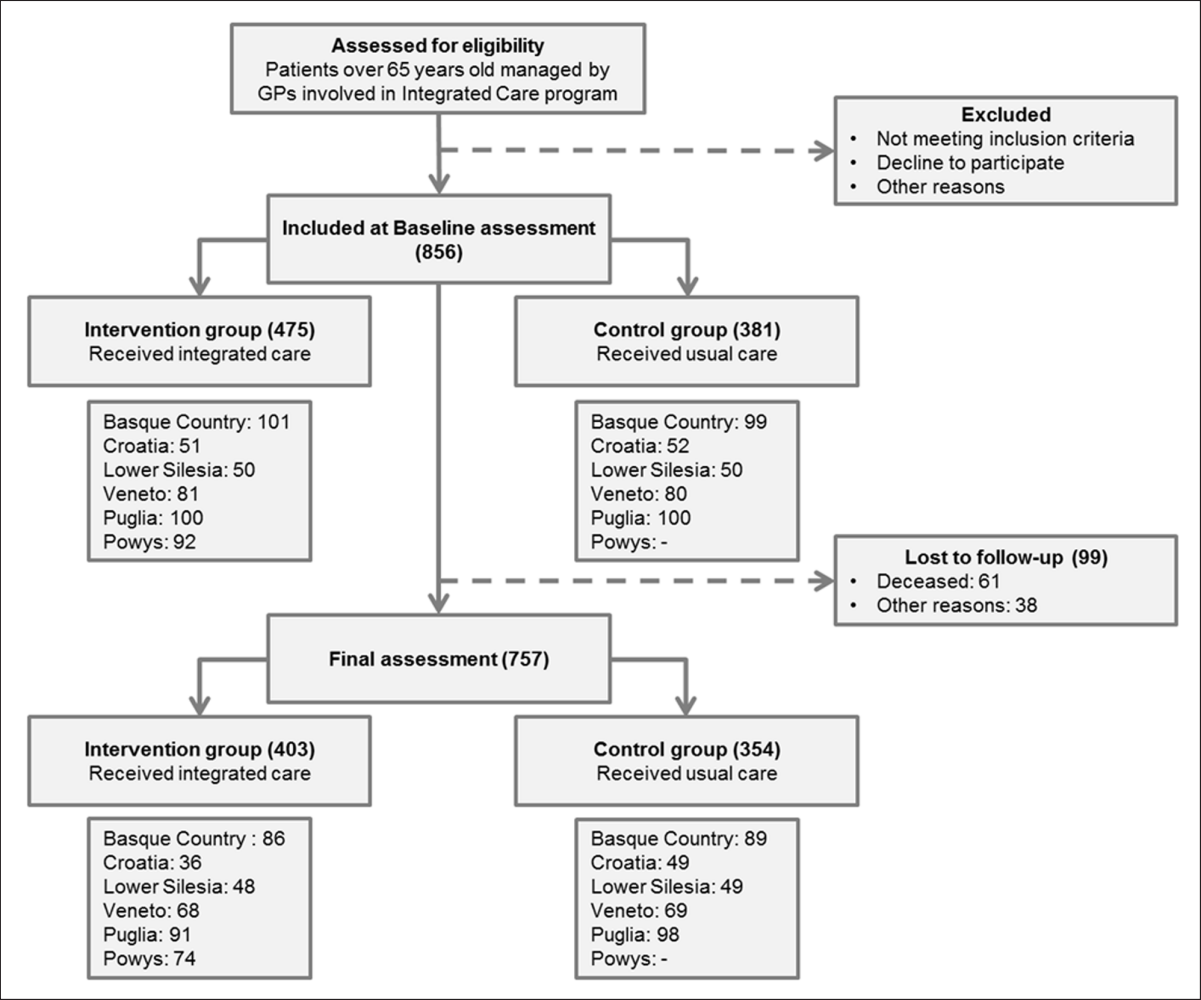
Diagram of the patients included. Flow of participants for control and intervention group, per site.

A total number of 856 individuals were recruited at the six pilot sites: 475 patients were assigned to the intervention group and 381 to the control group. Of the enrolled, 88% (84% and 93% in the intervention and control group, respectively; p = 0.081) were followed-up until the end of the observation period. Overall, 7% of the patients died during the study (8% in the intervention group and 6% in the control group; p = 0.124) and 4% were lost to follow-up (7% and 1% in the intervention and control group, respectively; p < 0.001). The flow chart is shown in Figure 1.

The baseline sociodemographic and clinical conditions are presented in Table 1. Among the participants, 51% were men, with a mean age of 77.6 years (SD: 7.7), and 43% had only primary level education. They presented a high degree of comorbidity (age-adjusted CCI: mean of 7.7; SD: 2.9), but conserved a high level of physical functional capacity (Barthel Index, median of 100). As explained earlier, HbA1c and creatinine levels were only collected for patients with specific diseases (ie, Diabetes and CHF). The baseline assessment showed some significant differences in lifestyle and clinical conditions between intervention and control groups. A higher level of education and more frequent use of personal computers were observed in the intervention group (p = 0.044 and p = 0.001, respectively). The mean values of BMI and the physical functional capacity were also statistically different (p = 0.004 and p = 0.003, respectively), although the differences did not reach clinical relevance.

**Table 1:** Baseline characteristics of the groups (intervention & control).

	Total	miss	Intervention	Control	p-value

Sample size	856		475	381	

Age	77.6 (7.7)	0	77.3 (7.7)	78.1 (7.6)	0.127
Gender (Female)	437 (51%)	3	252 (53%)	185 (49%)	0.182
Education		19			0.044
Lower than primary	129 (15%)		63 (14%)	66 (17%)	
Primary school	360 (43%)		187 (41%)	173 (46%)	
Secondary school	146 (17%)		84 (18%)	62 (16%)	
High school	130 (16%)		73 (16%)	57 (15%)	
College/University	72 (9%)		50 (11%)	22 (6%)	
Mobile use (Yes)	509 (60%)	5	287 (61%)	222 (58%)	0.501
Personal Computer use (Yes)	229 (27%)	8	147 (31%)	81 (21%)	0.001
Tobacco use		15			0.069
Never	473 (56%)		247 (54%)	226 (60%)	
Former	318 (38%)		181 (39%)	137 (36%)	
Current smoker	50 (6%)		34 (7%)	16 (4%)	
Body Mass Index	29.5 (5.7)	1	30 (5.9)	29 (5.4)	0.004
HbA1c	6.9 (1.1)	400	6.9 (1.2)	6.8 (1)	0.553
Creatinine	1.1 (0.5)	207	1.1 (0.5)	1.07 (0.5)	0.217
Barthel index, median (Q1, Q3)	100 (80,100)	31	95 (80,100)	100 (80,100)	0.030
Geriatric Depression Scale	4.1 (3.7)	6	4.1 (3.7)	4.1 (3.7)	0.800
Age-adjusted CCI	7.7 (2.9)	19	7.8 (3.1)	7.6 (2.7)	0.356
Comorbidity					
Myocardial infarct	160 (19%)	6	102 (22%)	58 (15%)	0.028
Congestive heart failure	528 (62%)	8	307 (65%)	221 (59%)	0.087
Peripheral vascular disease	324 (38%)	9	175 (37%)	149 (39%)	0.542
Cerebrovascular disease	208 (25%)	9	113 (24%)	95 (25%)	0.728
Dementia	91 (11%)	7	51 (11%)	40 (11%)	1.000
Chronic pulmonary disease	464 (54%)	1	266 (56%)	198 (52%)	0.286
Rheumatic disease	87 (10%)	6	45 (9%)	42 (11%)	0.507
Peptic ulcer disease	57 (7%)	7	30 (6%)	27 (7%)	0.715
Mild liver disease	88 (10%)	4	44 (9%)	44 (12%)	0.312
Diabetes without complication	432 (51%)	3	243 (51%)	189 (50%)	0.736
Diabetes with complication	193 (23%)	4	102 (21%)	91 (24%)	0.422
Hemiplegia or paraplegia	46 (5%)	3	29 (6%)	17 (4%)	0.379
Renal disease	202 (24%)	9	111 (24%)	91 (24%)	0.893
Any malignancy	90 (11%)	15	58 (12%)	32 (9%)	0.106
Moderate or severe liver disease	79 (9%)	7	46 (10%)	33 (9%)	0.724
Metastatic solid tumour	12 (1%)	17	11 (2%)	1 (0.3%)	0.025


Categorical data presented as frequencies and percentages (%) and continuous data as means and standard deviation, unless otherwise stated; CCI, Charlson Comorbidity Index; Comorbidity data show the incidence of comorbidity; miss, frequency of missing values; (Q1, Q3), Quartile 1 and 3; HbA1c and Creatinine only obtained for the patients reviewed to control specific diseases.

### The impact of the CareWell program

**Figure 2:**
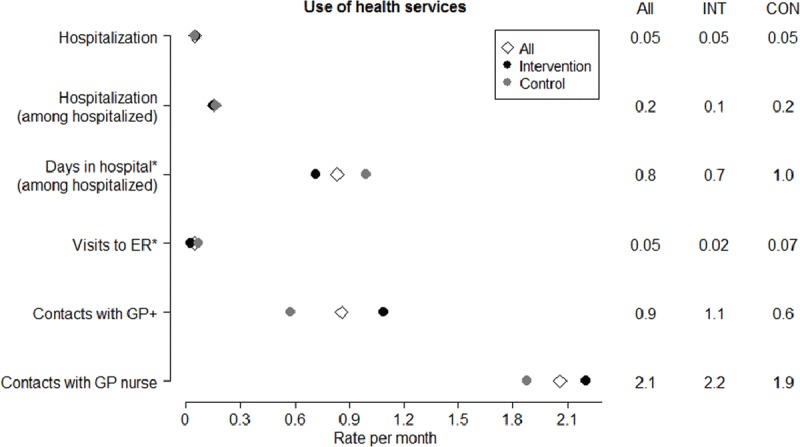
Use of health services. Data presented as mean rates of contacts per month. Mean values for all subjects (All), intervention group (INT) and control group (CON) are shown. Comparisons between the intervention and control groups were conducted by unadjusted and adjusted differences (adjusted by age, gender and age-adjusted Charlson Comorbidity Index). GP, General Practitioner; ER, Emergency rooms; * statistically significant difference (p < 0.05); + statistically significant difference (p < 0.05) only for unadjusted comparison.

Some differences were observed in health services use in the two assessed groups (Figure 2). The number of visits to ER services was significantly lower in the intervention group (p = 0.001). Remarkably, the number of visits to GPs and primary care nurses increased in that group; however, the latter lost its statistical significance when adjusted. Even though the hospitalization numbers were similar (the total mean rate per month was 0.05 (SD: 0.09) and 0.16 (SD: 0.09) for those with any hospitalization), the mean length of hospital stay among those who had been hospitalized was shorter in the intervention group (p = 0.033). No differences were observed for the use of social services [data not shown].

There was not a significant effect of the CareWell program on clinical outcomes (Table 2). Although BMI, and blood glucose were significantly reduced in the intervention group, the observed differences between groups were not statistically significant. Both groups showed an increase in the level of creatinine and a reduction in the value of Barthel Index, none of them reaching clinical significance.

**Table 2:** Basal and final results and differences between groups (intervention and control).

	Total	Differences per time point			Adjusted difference	

		Intervention	Control	p-value	CareWell Effect	p-value

**Clinical data**						

**Body Mass Index (BMI)**						0.099
*N*	*620*	*335*	*285*			
Basal	29.9 (5.4)	30.4 (5.8)	29.4 (4.9)	0.016	–	
Final	29.5 (5.4)	29.9 (5.6)*	28.9 (5)	0.038	–0.3 (–0.8,0.2)	
**Oxygen saturation (%)**						0.639
*N*	*546*	*261*	*285*			
Basal	95.8 (2.4)	95.4 (2.6)	96.2 (2.2)	<0.001	–	
Final	96.1 (2.3)	95.9 (2.1)*	96.2 (2.4)	0.114	0.1 (–0.4,0.5)	
**Blood glucose (mg/dl)**						0.677
*N*	*546*	*261*	*285*			
Basal	129.9 (45.9)	131.7 (50.8)	128.3 (41)	0.401	–	
Final	118.8 (50)	111 (48.7)*	123.2 (50.3)	0.012	–0.6 (–12.7,11.5)	
**HbA1c (%)**						0.131
*N*	*364*	*177*	*187*			
Basal	6.8 (1)	6.9 (1.1)	6.8 (1)	0.236	–	
Final	6.8 (1.2)	6.6 (1.1)	6.8 (1.2)	0.227	–0.4 (–0.8,–0.04)	
**Creatinine (mg/dl)**						0.182
*N*	*364*	*177*	*187*			
Basal	1.1 (0.4)	1.1 (0.5)	1.1 (0.4)	0.252	–	
Final	1.2 (0.6)	1.2 (0.6)*	1.1 (0.5)*	0.220	–0.1 (–0.1,0.02)	
**Geriatric Depression Scale**						0.233
*N*	*757*	*403*	*354*			
Basal	3.9 (3.7)	3.9 (3.7)	4 (3.7)	0.607	–	
Final	4 (3.8)	3.7 (3.7)	4.2 (3.8)	0.089	–0.6 (–1.6,0.3)	
**Physical functional status**						

**Barthel index**						0.889
*N*	*757*	*403*	*354*			
Basal	87.8 (20.3)	86.7 (22)	89.2 (18.2)	0.085	–	
Final	86.7 (21.4)	85.4 (22.9)*	88.1 (19.6)*	0.085	–1.1 (–3.2,1)	


Data presented as mean, standard deviation or their corresponding 95% confidence interval; N, sample size used in the analysis (only included pilot sites which has the follow-up for the corresponding parameter); * indicates if there are pre-post differences within each arm (intervention or control); CareWell effect, indicates the difference between the intervention over the control group, by the interaction coefficient between the care group and the follow-up period. The effect coefficient was estimated considering follow-up of 12 months, and the mixed-effect models were adjusted using age, gender, and age-adjusted Charlson Comorbidity Index.

**Figure 3:**
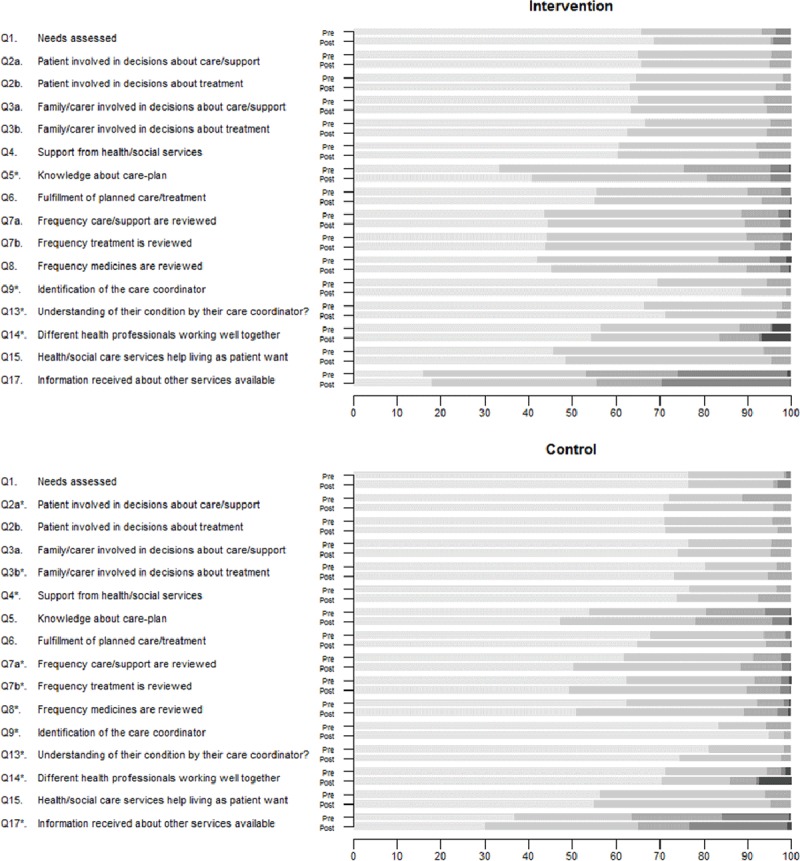
Patient’s satisfaction assessed by PIRU questionnaire: a) for intervention group and b) for control group. Data represents percentage of each category; color intensity indicates the level of satisfaction; the lighter the gray, the more satisfied are the participants; the darkest color indicates: “Don’t Know/not sure” for questions 1, 9 and 14; pre-post comparisons were performed for each group; * statistically significant difference (p < 0.05).

Finally, patient’s satisfaction before and after the intervention, measured using PIRU questionnaire, is presented in Figure 3. For each question, the pre-post differences for intervention and control groups are shown. The color intensity reflects the level of satisfaction, the lighter the gray is, the more satisfied are the participants. The darkest color indicates: “Don’t Know/not sure” for questions 1, 9 and 14. Overall, the results showed a high level of satisfaction in both groups at baseline and after follow-up. Satisfaction levels were slightly higher in controls than in the intervention group. Nevertheless, over time, a larger effect in the intervention group was observed. Some of the questions showed a significant improvement in the intervention group: the level of the care-plan information received by the patients was higher (Q5), the patients were more aware of the role of care coordinators (Q9), and the feeling that these professionals understood the patients and their condition was strengthened (Q13). In the control group, there was a significant improvement in the identification of the care coordinator (Q9); however, the patients felt that the understanding of their condition diminished over time (Q13). Patients in that group expressed also significantly reduced satisfaction from baseline with the involvement level of their families with the decision-making process (Q3b) and the level of support received from the health and social institutions (Q4). Moreover, they became less satisfied with the review of their care and support (Q7a), treatment (Q7b), and medication (Q8). Finally, patients in both groups felt that there was insufficient information about other services available to someone with the similar condition (Q17); during the follow-up, this perception strengthened in the control group. Finally, notion about different health professionals working well together (Q14) decreased in both groups.

## Discussion

Integrated care has been identified as one of the six specific priority actions of the European Innovation Partnership on Active and Healthy Ageing [25]. Here, we present an assessment of the impact of an integrated healthcare intervention targeting aged complex patients with multimorbidity piloted in six European regions, based on improvement actions around eight dimensions in care coordination and twelve technological adaptations. Specific changes were based on self assessment and local priorities.

The main outcome of the CareWell program was observed in the use of health services, where a change in the service use pattern was observed. Patients receiving the CareWell integrated care intervention improving care coordination and patient empowerment and home support, decreased the number of visits to ER, had shorter hospital stays, and used more primary care services. The observed changes support the key role of primary care in integrated care programs [7]. A reduced number of reviews of integrated care programs for chronically ill patients, mention effects on hospitalization and decreasing length of stay, with statistically significant differences, compared to the usual care group [14].

The intervention did not produce strong clinical effects, in agreement with other similar studies [26,27]. The BMI, and blood glucose were significantly reduced in the intervention group during the follow-up period, but without significant differences between intervention and control groups. The short extent of the observed clinical effects might be due to the short follow-up period and to the high degree of complexity of the assessed patients. It would be worthwhile to increase the follow-up period to re-assess the potential clinical effects of this approach. Regarding presence of depressive symptoms, no differences were found in the GDS. This finding is in line with a systematic review about comprehensive care programs [16]. That study also indicates that there is no evidence for improvements of physical functional status by this type of care programs.

The patients showed a remarkably high satisfaction level. At baseline, it was higher in the control group than in the intervention group. However, more improvements were reported in the intervention group. They felt that the information sharing, coordination, and participation had improved throughout the care process, as had the identification of the care coordinator and the understanding of their condition by this named professional. Consequently, they felt empowered and firmly placed at the center of the care program. The European ICARE4EU project, which aims to disseminate knowledge of European integrated care programs addressing multimorbidity [28], suggested that patient empowerment and education needs more attention when implementing patient-centered integrated care for multimorbidity patients. Our results indicated that CareWell program put an effort in informing patients and supporting their self-management skills.

The fragmented organization of healthcare delivery is inappropriate for multimorbid patients, who account for the highest share of preventable hospitalizations and for most of the healthcare expenditure [29]. The design and implementation of integrated care interventions in care coordination and patient empowerment, based on local self assessment, and decision making, but using a common framework could offer an alternative, feasible and more adequate way to manage these patients [30]. This work examines the effect of the implementation by studying a large range of domains, such as, the patient’s satisfaction, use of health resources, clinical outcomes, and physical functional status, from a quantitative evaluation. Future research will analyzed the organizational aspects and user’s and professional’s perspective from a qualitative approach obtained in the project.

Based on all the mentioned results and in the experience achieved by the implementation of the program in the six specific pilot sites along the project duration, CareWell project provide insights in how to implement an integrated care program for complex patients with multimorbidity in order to ensure the transferability [31].

CareWell project has suggested that technology-enabled integrated care must be recognised as a complex service innovation and can be fully effective through change strategies that influence, educate, train and enable transformation in the way care professionals work and how they engage effectively with patients and carers [32]. The impact of CareWell, considered as the the long term consequences of the Project are difficult to quantify. A budget impact analysis was performed for a specific area in the Basque Country pilot site within the CareWell project [33]. This allowed to ascertain the economic impact of the intervention which provided a comprehensive understanding of the real impact of the implemented integrated care model. Furthermore, the study has derived in a framework which could be used not only for the Basque Country, but for the rest of sites. Moreover, CareWell was included amongst the top three projects with regards to overall impact after the in depth analysis produced for EU-Funded Research and Innovation on ICT for Active and Health Ageing, covering the fall prevention; better connected through integrated care and robotics for ageing well [34].

The main limitation of this project was the method of allocation considering that random group assignment was not possible, and this could affect the interpretation of the results found. Nevertheless groups were comparable except for the level of education and electronic proficiency. Moreover, the potential biases produced by this difference were controlled by adjusting by age, gender and degree of comorbidity because in the final models education and electronic proficiency showed no significant effect. Also the large number of participants has to be considered.

It is also important to note that there have been differences in the length of follow up between intervention and control group. However, it has been suggested that, as a rule of thumb, a drop-out rate less than 5% poses little challenge to the results [35]. The variability in the implementation of the intervention through the sites needs to be discussed. The actual intervention in each site was dependent on their baseline situation and on the local context and priorities. The number of stakeholders involved, the coordination procedures, performed functions, and communication channels varied substantially. The extent to which the innovative approaches to chronicity can be deployed depends on the level of maturity of the studied health systems. However, all the six models were based on care coordination between healthcare professionals at different care levels, remote and proactive patient monitoring, and patient/caregiver empowerment; all of these were facilitated by the use of ICTs. Furthermore, the six integrated care models shared the key components of the intervention, such as procedures to activate the primary or community care after discharge from the hospital, close monitoring of patients at home by care managers, and the provision of structured patient empowerment programs. ICT applications may provide new and effective tools to promote information gathering and communication, but the effectiveness of interprofessional collaboration will always depend more on social relationships and on the context of the organization within which they are placed [36]. The heterogeneity of the models, far from being a limitation, allows future implementation and transferability of the program by adapting it to the characteristics and needs of the particular health system. This helps to avoid potential failures due to methodological issues. The methodology used here makes it possible to control for the variability introduced by the high heterogeneity between the setting and organization models, including the sites as random effect in the performed generalized mixed models. Moreover, the results of the study suggest that the inclusion of some key elements of the new integration care model derived in a relevant impact in the provided care [37].

## Conclusion

In Europe, the number of older people with multiple chronic conditions is increasing. New strategies, such as integrated and technology-supported care programs, are needed to address the complex health and social needs of these patients and their carers. The CareWell program is an integrated care approach specifically designed to manage these patients. Its implementation suggested improvements in the use of health services with a shift towards primary care services, some clinical improvements as well as a better patient empowerment and understanding.

## Trial Registration

ClinicalTrials.gov Identifier – NCT03042039

## Additional File

The additional file for this article can be found as follows:

10.5334/ijic.4711.s1 Table S1. Description of the existing organizational model, and the specific elements of the integrated care model and its implementation by each pilot site.
