# Supplementary material for: Impact Assessment of an Innovative Integrated Care Model for Older Complex Patients with Multimorbidity: The CareWell Project
Source: Int J Integr Care. 2020 May 22;20(2):8. doi: 10.5334/ijic.4711 (PMC7243835; doi:10.5334/ijic.4711)
Supplement: Table S1. — Description of the existing organizational model, and the specific elements of the integrated care model and its implementation by each pilot site. [file ijic-20-2-4711-s1.pdf]

Table S1: Description of the existing organizational model, and the specific elements of the integrated care model and its implementation by each pilot site.

	Pilot site					
	**Basque Country (Spain)**	**Zagreb (Croatia)**	**Lower Silesia (Poland)**	**Veneto (Italy)**	**Puglia (Italy)**	**Powys (Wales)**
**Setting**						
Population (inhabitants)	2,200,000	1,100,000	3,000,000	4,900,000	4,100,000	133,000
**Existing organization model**						
**Commonalities**						
Stable patient:						
GP and GP nurse responsible for case management	✓	✓	✓	✓	✓	✓
Social workers in charge of social care interventions thought home visits	✓	✓	✓	✓	✓	✓
Unstable patient (out of hospital):						
GP (with/without GP nurse) defines therapeutic care plan, follows up the patient´s status, and refers to specialist if necessary.	✓	✓	✓	✓	✓	✓
Can activate the emergency services	✓	✓	✓	✓	✓	✓
Within hospital care:						
Specialists are in charge of diagnosis, drug prescription and therapeutic plan definition	✓	✓	✓	✓	✓	✓
Hospital nurse coordinates tests, provides medication (prescribed by the specialist), implements therapeutic plans	✓	✓	✓	✓	✓	✓
At hospital discharge:						
GP and GP nurse responsible for patient follow-up and training to promote self-management.	✓	✓	✓	✓	✓	✓
ICT tools						
Community alarm	–	–	–	–	–	–
**Differences**						
Stable patient:						
Identification of frail patients by risk stratification tools	✓	✓	–	✓	✓	–
Coordination between health and social care	✓	–	–	✓	–	–
Face-to-face multidisciplinary team meeting	✓	✓	–	✓	–	✓
Coordination of healthcare resources	✓	–	–	✓	–	–
Unstable patient (out of hospital):			–			
Can activate home care/home-hospitalization nurses	✓	–	–	✓	–	–
Within hospital care:			–			
Referent internist: case management and coordination with GP	✓	–	–	✓	–	–
At hospital discharge:						
Hospital liaison nurse: communication between primary and secondary care	✓	–	–	✓	–	–
GP and GP nurse can schedule home	✓	–	–	✓	–	–

	Pilot site					
	**Basque Country (Spain)**	**Zagreb (Croatia)**	**Lower Silesia (Poland)**	**Veneto (Italy)**	**Puglia (Italy)**	**Powys (Wales)**
visits for an intensive follow-up						
ICT tools						
Electronic prescription	✓	✓	–	✓	–	–
Messaging between professionals	✓	✓	–	✓	–	✓
Resource and process management platform	✓	–	✓	✓	–	–
Health and wellbeing information portal	–	–	–	–	–	✓
Telehealth, Telecare	✓	–	–	✓	✓	–
**New Integrated care model**						
**Integrated care coordination**						
Stable/Unstable patient:						
Improved definition of care manager role	–	–	–	–	✓	–
Improved information sharing among healthcare professionals via central storage of data and definition of shared care plans	✓	–	–	–	✓	✓
Within hospital care:						
Wider deployment of reference internist	✓	–	–	–	–	–
At hospital discharge:						
Wider deployment of hospital liaison nurse	✓	–	–	–	–	–
Smooth transition support by facilitating information sharing, using ICT systems	–	–	✓	–	–	✓
ICT tools:						
Improved communication between healthcare professionals	✓	✓	–	✓	✓	✓
Interconsultations via EHR	–	–	–	✓	–	–
Video consultations	–	–	–	✓	–	✓
Virtual space within EHR	–	–	–	–	✓	–
ICT system integration	–	✓	–	–	–	–
**Patient empowerment and home support**						
ICT tools						
Promotion of patient and caregiver empowerment through access to the health-related educational material	✓	✓	–	✓	–	–
Patients can access or enter clinical information and book appointments	✓	–	–	✓	–	✓
Messaging between healthcare professionals and patients/caregivers	✓	–	–	–	–	–
Monitoring of patient health status, mainly led by nurses	✓	✓	✓	✓	✓	✓
Remote monitoring (telemonitoring)		✓	✓	✓	✓	✓
Phone calls	✓	–	–	–	–	–
Supervised questionnaires online	✓	–	–	–	–	–
Teleconsultations	–	–	–	–	✓	–

GP, general practitioner; EHR, electronic health record; ICT, information and communication technology.

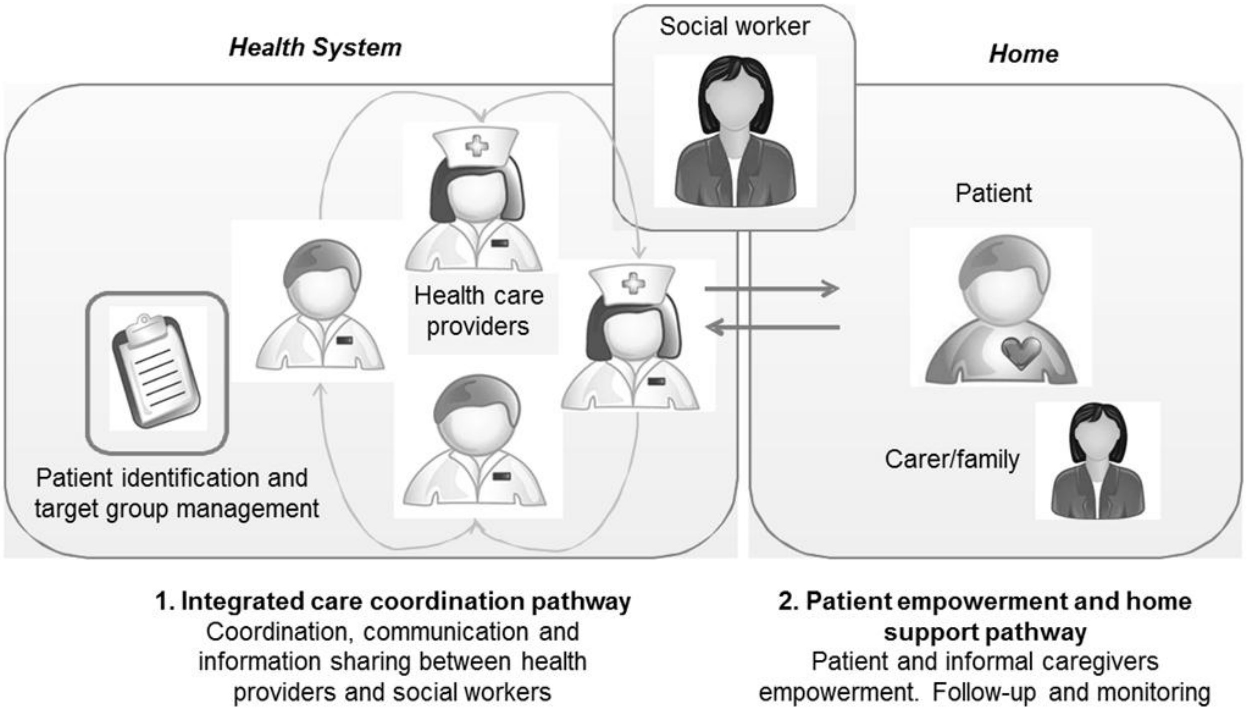

Fig1 Additional Description of the integrated care pathway. The program is based on two main elements, integrated care coordination and patient empowerment and home support pathways
